# iDog: an integrated resource for domestic dogs and wild canids

**DOI:** 10.1093/nar/gky1041

**Published:** 2018-10-29

**Authors:** Bixia Tang, Qing Zhou, Lili Dong, Wulue Li, Xiangquan Zhang, Li Lan, Shuang Zhai, Jingfa Xiao, Zhang Zhang, Yiming Bao, Ya-Ping Zhang, Guo-Dong Wang, Wenming Zhao

**Affiliations:** 1 BIG Data Center, Beijing Institute of Genomics, Chinese Academy of Sciences, Beijing 100101, China; 2 University of Chinese Academy of Sciences, Beijing 100049, China; 3 State Key Laboratory of Genetic Resources and Evolution, Kunming Institute of Zoology, Chinese Academy of Sciences, Kunming 650223, China; 4 CAS Key Laboratory of Genome Sciences and Information, Beijing Institute of Genomics, Chinese Academy of Sciences, Beijing 100101, China; 5 Center for Excellence in Animal Evolution and Genetics, Chinese Academy of Sciences, Kunming 650223, China

## Abstract

The domestic dog (*Canis lupus familiaris*) is indisputably one of man's best friends. It is also a fundamental model for many heritable human diseases. Here, we present iDog (http://bigd.big.ac.cn/idog), the first integrated resource dedicated to domestic dogs and wild canids. It incorporates a variety of omics data, including genome sequences assemblies for dhole and wolf, genomic variations extracted from hundreds of dog/wolf whole genomes, phenotype/disease traits curated from dog research communities and public resources, gene expression profiles derived from published RNA-Seq data, gene ontology for functional annotation, homolog gene information for multiple organisms and disease-related literature. Additionally, iDog integrates sequence alignment tools for data analyses and a genome browser for data visualization. iDog will not only benefit the global dog research community, but also provide access to a user-friendly consolidation of dog information to a large number of dog enthusiasts.

## INTRODUCTION

The domestic dog (*Canis lupus familiaris*) and humans have enjoyed one of the oldest comraderies in history (1), together concurring several biotic and abiotic odds to settle in all corners world. The release of dog genome sequence in 2005 (2) and rapid advances in high-throughput sequencing technologies in recent years have greatly accelerated studies on dogs, especially about its domestication and evolution (3–12). To further cement their participation in human life, the domestic dog has emerged as a premier model for heritable human diseases, facilitating the identification and study of numerous disease loci (13–16), particularly those associated with cancers. For instance, Sara Mata López used a 5-month-old male Border Collie dog with progressive muscle weakness and dysphagia as the model for Duchenne muscular dystrophy to investigate pathogenesis and preclinical therapy (17). In 2015, the International Dog10K Genomes project (http://dog10kgenomes.org) was launched, aiming at sequencing 10 000 genomes covering as many dog breeds as possible plus other canid species across the world, and provide a comprehensive resource for the canine community involving an enormous amount of omics data. With the increasing volume of various types of omics data, constructing a comprehensive platform specializing in domestic dog for data archiving, interrogation, online analyses and visualization is necessary.

Compared to the popularity of dog studies, relevant data resources are rare. In the year 2015, we published the first canine-dedicated SNP database, DogSD (18), that focuses on genomic variations. The Inherited Diseases in Dogs (IDID; https://www.vet.cam.ac.uk/idid) (19), Canine Inherited Disorders Database (CIDD; http://cidd.discoveryspace.ca) (20), and Online Mendelian Inheritance in Animals (OMIA; http://omia.org) (21) databases focus specifically on inheritable disease of dogs. IDID is no longer operational. CIDD provides detailed descriptive information of canine inherited disorders in various breeds, while OMIA curates genes and variants information related to dog diseases from published papers. However, these two databases have different disease description formats and disease-naming methods, and are not linked to each other to provide convenience in searching for a particular dog disease and obtaining its detailed description and associated genes. On the other hand, there are up to 400 domestic dog breeds (10) and many international dog clubs, such as American Kennel Club (AKC; http://www.akc.org), Canadian Kennel Club (CKC; https://www.ckc.ca/en), United Kennel Club (UKC; https://www.ukcdogs.com), Federation Cynologique Internationale (FCI; http://www.fci.be/en), and China Kennel Union (CKU; http://www.cku.org.cn), have gathered enormous phenotypic data of numerous dog breeds, but their naming standards and descriptive data are not unified. For instance, a breed called Soft Coated Wheaten Terrier in AKC is called Soft-coated Wheaten Terrier in CKC and UKC, but Irish Soft Coated Wheaten Terrier in FCI. This is can be very confusing to users. Moreover, the disease and phenotype information of breeds are still isolated in all the above-mentioned resources. Since domestic dog is a leading example of extreme phenotypic diversification under domestication, development of an inclusive database integrating phenotype, genotype, disease, breed information and other important knowledge tips be a great boost to the dog research community.

Here, we present iDog (http://bigd.big.ac.cn/idog), a comprehensive data resource with online data analysis tools to provide public and free data service for scientific research. Currently, iDog includes two recently released genomes (22), genomic variations, phenotype/disease traits, gene expression profiles, functional annotations, gene homologs and informative literature.

## IMPLEMENTATION

iDog is implemented using the J2EE framework, MySQL (http://www.mysql.org; a free and popular relational database management system) and Apache Tomcat Server (http://tomcat.apache.org; an open source software implementation of Java Servlet and Java Server Pages). Web user interfaces are developed using JSP (JavaServer Pages; a technology facilitating rapid development of dynamic web pages based on the Java programming language), HTML5, CSS3, AJAX (Asynchronous JavaScript and XML; a set of web development techniques to create asynchronous applications without interfering with the display and behaviour of the existing page), JQuery (a cross-platform and feature-rich JavaScript library; http://jquery.com, version 3.2.1) as well as BootStrap (an open source toolkit for developing web projects with HTML, CSS and JS; https://getbootstrap.com, version 3.3.7). For dynamic data visualization, ECharts (a declarative framework for rapid construction of web-based visualization; http://echarts.baidu.com, version 4.1.0) is incorporated to generate charts, GBrowse_syn (23) is used for genome synteny visualization. Additionally JBrowse (24) and GBrowse (25) are adopted for chromosome-based data visualization.

## DATABASE CONTENTS AND FEATURES

**Figure 1.**
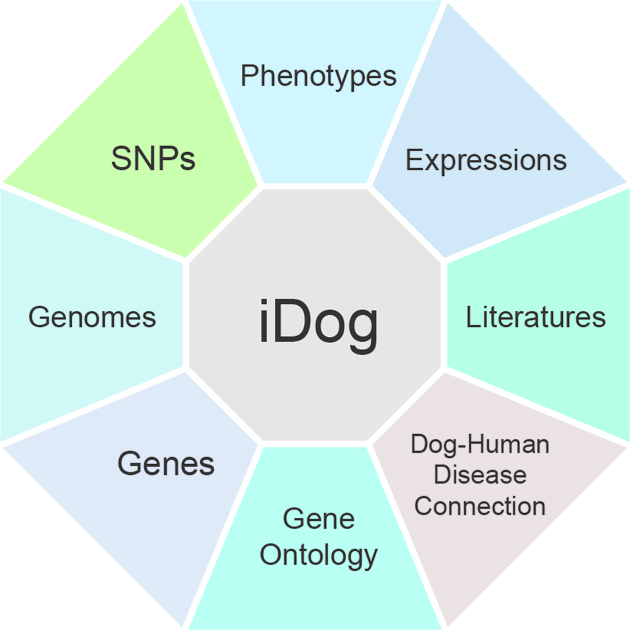
Data resources in iDog. In the current version, iDog integrates various data types including: (i) phenotype data with breeds, diseases, and G2P pairs information; (ii) expression data of gene profiles from RNA-seq projects; (iii) a collection of disease-associated literature; (iv) Dog–Human Disease connection with homologous genes information of 10 organisms especially in dog and human; (v) Gene Ontology with genes annotated in GO terms; (vi) gene as a data hub links to other data types; (vii) genome data of two *de-novo* genome assemblies with annotated information; (viii) SNP data of 127 dog samples including non-redundant SNPs as well as individual SNPs.

Currently, iDog hosts various data types including genomic data of new *de-novo* genome assemblies with well annotated information and SNP data of resequenced samples, expression data from published RNA-Seq projects retrieved from NCBI, and phenotype/disease traits from public resources, gene homolog of multiple organisms and literature on dog diseases. The latest boxer genome assembly (canfam3.1) (2) was used as the reference genome to analyse the genomic data, with the annotation file downloaded from Ensembl (26). Figure 1 shows the data type curated in this database and the data statistics information are summarized in Table 1. In order to identify gene functions, Gene Ontology (GO) (27) and its annotation information was organized and imported into the database. In addition, some commonly used data analysis and visualization tools, such as Blast (28) and GBrowse_syn (23), are included to provide free access data visualization services to users.

**Table 1.** Data statistics of iDog (as of 17 September 2018)

Data contents	Data statistics
**Gene**	
Gene integrated from Ensembl database	32 220
Gene associated with Dog disease	229
**SNP**	
Individuals	127
Non-redundant SNPs identified	42 871 184
Non-redundant SNPs annotated in gene	22 031 720
**Phenotype & disease**	
Standard Breeds	473
Diseases	783
Genotype–phenotype pairs (G2Ps)	594
**Genome**	
Scaffolds for wolf	581
Scaffolds for Dhole	749
**Gene expression**	
RNA-Seq projects	7
Experiments	83
Tissues	5
Genes well annotated	27 534
**Gene ontology (GO)**	
Molecular function	
Genes	14 361
Annotations	60 030
Biological process	
Genes	15 079
Annotations	103 120
Cellular component	
Genes	15 884
Annotations	65 214
**Literature**	
Papers and books	6 535

### Gene

**Figure 2.**
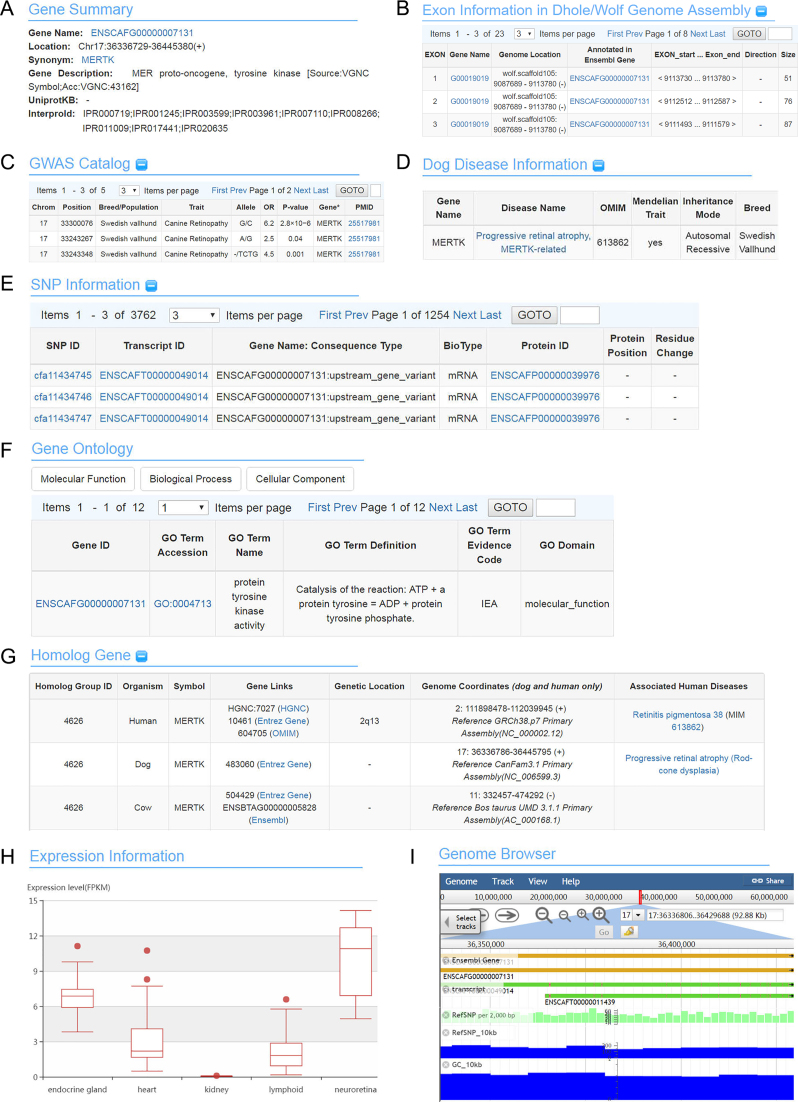
Screenshots of a gene report page for *MERTK*. (**A**) The summary information of gene. (**B**) The exon with annotated information of the predicted gene. (**C**) G2P pairs information about this gene. (**D**) Associated diseases information from the phenotype data. (**E**) SNPs annotated in the gene. (**F**) Gene Ontology information. (**G**) Homologous genes information in 10 organisms. (**H**) Gene expression profiles in five tissues. (**I**) Genome browser for visualizing different tracks.

Gene database houses genes and annotation information was filtered by Ensembl BioMart (http://www.ensembl.org/biomart/martview/) (29) using the Canfam3.1 gene sets with both basic and external attributes, like Gene ID, Gene Name, GO term, Interpro ID and UniPortKB ID. In total, 32 220 genes with unique ‘ENSCAFG’ Gene IDs were obtained. These genes were used as a ‘data hub’ to link all data types mentioned above through the ‘ENSCAFG’ Gene IDs. Integrated information of each gene, such as the basic information, gene structure, and associated SNP information, are shown on one page to allow easy browsing (Figure 2).

### Phenotype

Dog Phenotype Database (DogPD) integrates phenotypic data including breeds, diseases, and genotype–phenotype (G2P) pairs. The breeds information was collected from AKC, CKC, UKC, FCI and Wikipedia. Initially, original breeds information was adopted from public websites, including 263 from AKC, 210 from CKC, 374 from UKC, 343 from FCI and 513 from Wikipedia. We defined a few rules to standardize breed names by considering the character, origin of the dog, as well as the popularity of the names. We also defined controlled vocabularies to normalize the description of characters. For instance, the word Wire-Haired was used to represent Wire, Wire-haired, Wirehaired, Coarse-Haired, Coarse Haired, Rough-Haired, Rough Haired and Rough terminologies. Based on these rules, we combined all these data, normalized both the breed names and the description of characters, and masked any redundant information. Finally, 473 non-redundant breeds with unique names were acquired and uploaded onto the database, and a unique number assigned to each breed for easy identification. Similarly, to organize the diseases data, original information was adopted from CIDD, OMIA and TheDogPlace (http://www.thedogplace.org). After curation and merging, 783 items were associated with particular dog breeds, with their gene information acquired and uploaded onto the database, together with the relationship among breeds and diseases with their associated genes built automatically. In total, there are 216 breeds associated with 420 dog disease items in addition to 229 dog disease associated genes. In order to help dog owners and veterinarians understand or manage dog diseases more effectively, the dataset contains detailed information integrated from different sites covering disease description, symptoms, causes, inheritance mode, diagnostic methods, treatment, and even advisory tips to veterinarians or breeders. In total, 622 items with high-quality disease information are currently available in the iDog database. A little different from the breed and disease data curation strategies, for G2P pairs, we acquired data from Genome Wide Association Study (GWAS) papers published available in PubMed from 2010 to 2018, and a total of 594 G2P pairs were obtained and included in the dataset.

**Figure 3.**
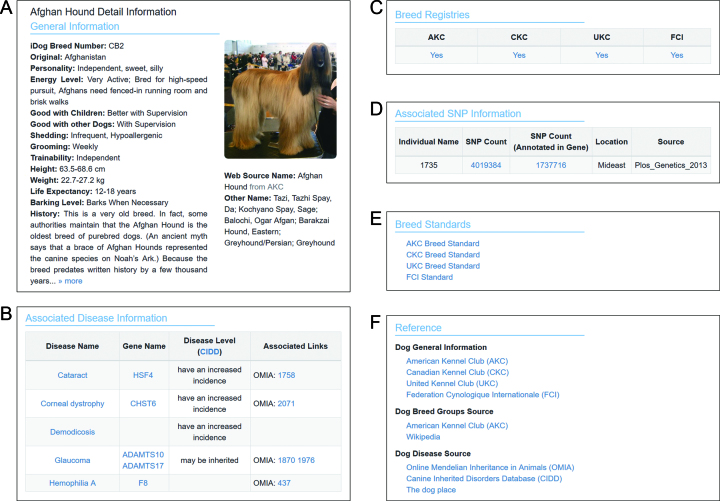
Screenshots of phenotype database. (**A**) General information about a breed curated from public resources. (**B**) Associated disease information of this breed including disease name, gene name, and disease level information integrated from OMIA and CIDD. (**C**) The breed registration in international kennel clubs. (**D**) The individual SNP information of this breed. (**E**) The breed standard in international kennel clubs. (**F**) The data sources for this breed.

DogPD provides a user-friendly web interface for users to browse and retrieve information. For instance, on the Breed Page, users can browse breed data in three ways: the indexed first letter of breed name from A to Z, the group/kind of breed which is termed as ‘Breed Groups’ in the website, and the origin of the breeds. Users can also filter their best-loved breeds using several keywords including breed name, temperament, size, weight, height, life expectancy, trainability, grooming, and shedding. In order to improve the readability of the integrated data, detailed information about breeds were divided into six categories: general information, the links of breed registries in international kennel clubs, associated diseases, associated SNPs, the breed standard, and the reference (Figure 3). On the Disease Page, several optional search textboxes are included to enable convenient retrieval of results by keyword, and on the detailed page of a specific disease, the associated diseases in CIDD/OMIA are listed automatically along the associated breed lists.

### Genome

Dog Genome Database (DogGD) aims at providing a public warehouse to release, annotate, and update whole genome sequences of dogs. Currently, DogGD hosts two *de-novo* genome assemblies, dhole and gray wolf (22), contains the genomes with well annotated genes, structural variations, and results of a comparative analysis of the two individuals. Additionally, some statistical data are included, for instance, the N90 and the distribution of scaffold sequence length. In DogGD, the scaffold length >500kb was selected to parse and display results. In total, DogGD hosts 749 scaffolds of the dhole genome, 581 scaffolds of the wolf genome, 18 077 predicted genes in the dhole, 18 172 predicted genes in the wolf and annotations generated in Ensembl and UniProt (30) (Swiss-Prot and TrEMBL).

DogGD provides browser views for sequence, annotation data, comparative genome analysis result, and statistical information about repeat sequences. For instance, the scaffolds list shows structural information with scaffold length, annotated gene number, aligned reference length and coverage with respect to the reference. From this table, users can have a general overview of all the data in its entirety. In order to make different tools compatible, DogGD provides two formats to show detailed gene information, the ‘Custom’ and the ‘Genbank’ formats, which allows users to read or save data in their local machines. In addition, DogGD incorporates the NCBI Blast tools (v2.6.0, https://blast.ncbi.nlm.nih.gov/Blast.cgi) (28) for online data analysis. Users can upload their sequence or sequence file to perform blastn, blastp, blastx or tblastn against whole genome sequences, CDS regions, or peptides. Moreover, a visualization tool GBrowse_syn (23) can be used to show the genome synteny between dhole and wolf.

### Genomic variation

Dog SNP Database (DogSD) houses high quality single nucleotide polymorphism (SNP) data of domesticated dogs and gray wolves. The current DogSD integrated in iDog is an updated version of what was previously released in 2015. It contains 127 samples in total from four previous studies (5,31–33) and dbSNP (version 146). There are 62 new compared with the first version. We annotated SNPs in the genomes using the Ensembl Variant Effect Predictor (VEP) tool (34) (ftp://ftp.ensembl.org/pub/release-84/variation/VEP/) with a parsed VCF files. Allele frequency was calculated for wolf/dog populations for each SNP for the 127 samples. As a result, 42 871 184 non-redundant SNPs were identified and uploaded onto the database.

DogSD provides an easy-to-use interface for accessing all the genomic data and integrates JBrowse for data visualization. Users can access either non-redundant SNPs or each individual's SNPs and view SNPs annotated in genes, all organized by chromosome. A search page with genomic location, consequence type, and the associated gene is provided for users to search an individual's SNPs. Moreover, comparative analyses between two or more individuals can be easily done through the search engine, while compiles detailed information that can be downloaded. All the raw sequence data can be found in the Genome Sequence Archive (35) database of the BIG Data Center (36,37).

### Expression

Dog Expression Database (DogED) integrates genome-wide gene expression profiles derived from RNA-Seq data analysis of various dog tissues. We retrieved the RNA-Seq projects of *Canis lupus familiaris* from NCBI BioProject (https://www.ncbi.nlm.nih.gov/bioproject/browse) (38) and masked datasets with no publications to ensure high data quality. We then used SRA Toolkit (v2.7.0) to convert the data format to FASTQ, and FASTQC (v0.11.7) combined with cutadapt (v1.16) (39) to do quality control (e.g. base quality > 20, remove adaptors) and remove low quality reads. Then, we mapped the high-quality reads to the dog reference genome (Canfam3.1) using HISAT (v2.2.1) (40), filtered samples and data with low mapping rates (<70%), and adopted FPKM (fragments per kilobase of exon per million fragments mapped) tool to estimate expression levels of genes and transcripts with the help of RSEM (v 1.3.0) (41). Meanwhile, differentially expressed genes in multiple samples within one BioProject were identified using RSEM. In the end, seven high-quality RNA-Seq projects were selected, including 83 experiments, covering five tissues and yielding 27 534 gene expression profiles, with at least one group of differential gene expression profile provided in each project.

DogED provides a user-friendly web interface to enable easy access, search, and view of gene expression profiles. Users can filter the gene expression profiles by gene name, genome location, GO, InterPro protein domain, and FPKM value. For a given gene, the expression profile for all the five tissues are visualized in a box plot. Users can filter the differential gene expression profiles within one project by gene id/symbol, up or down regulated status, log_2_ fold-change value and *P* value. For a differentially expressed gene, its expression comparison across multiple samples can be visualized in a heatmap plot. The whole gene expression profile files and the gene expression files of each BioProject can be downloaded from the FTP server.

### Functional annotation

Function Annotation is used to identify gene function. Currently, iDog integrates GO and associated dog gene annotation. We downloaded the GO database from Gene Ontology Consortium (http://www.geneontology.org/) (27) and genes with GO annotations from Ensembl by using the BioMart (29) tool. We retrieved each term of GO based on the criteria that, if one gene is annotated into a GO term then it needs to be annotated into its parent GO term. As a result, 14 361 genes with 60 030 annotations in Molecular Function, 15 079 genes with 103 120 annotations in Biological Process, 15 884 genes with 65 214 annotations in Cellular Component were identified.

GO is displayed using a tree plugin, whereby unfolding the GO tree by clicking one term, the number of corresponding genes and annotations will be shown. In addition, a GO search function is provided for users to filter GO items by accession or term, and a result of terms will be listed. By clicking one term in the result, a GO tree will be opened indicating the location of this term.

### Literature

The main purpose of Literature is to provide a public literature repository and collection of various research studies on domestic dogs. Currently, literature mainly focuses on dog diseases. The abstracts of literature were fetched from PubMed (http://www.ncbi.nlm.nih.gov/pubmed) (38) using NCBI’s E-utilities and PubMed ID. The relationship between given piece of literature and disease was built according to some keywords, such as the disease name. In total, 6535 papers/books are included and 577 diseases linked to them accordingly.

### Dog-Human disease connection

Dog-Human Disease Connection is used to identify homologs of dog genes in other organisms especially human. Homolog gene information was collected from NCBI HomoloGene (https://www.ncbi.nlm.nih.gov/homologene) (38), and 10 organisms including dog, human, chimpanzee, Rhesus monkey, cattle, mouse, Norway rat, chicken, tropical clawed frog and zebrafish were used to filter the homolog gene information. Disease-associated genes from DogPD were then used as an input to filter and get the final list of homologous genes in these 10 organisms. In order to understand possible phenotype/disease traits in humans, related OMIM (42) data was also curated and integrated. As a result, 213 dog homolog genes associated with human diseases were included in the database.

### Online tools

iDog provides an open and free platform for users to analyze their data using integrated online tools. The system infrastructure is built on a highly available and flexible cloud platform, namely BioCloud (http://biocloud.big.ac.cn), which enables users to create applications/pipelines, upload and analyze their own data, access, and visualize results online. Users only need to upload data, fill in some parameters and submit the task, then a results page with ‘task running’ status will appear. If an email address is filled, an email notification will be sent automatically when the task is submitted or finished. For a better understanding of the usage of each tool, a sample data page and corresponding results page are provided. Currently, six online tools are running in iDog, four for data visualization and two for sequence alignment.

## DISCUSSION AND FUTURE PLANS

iDog is dedicated to a comprehensive integration of omics data of domestic dogs. The current implementation of iDog integrates important data including phenotype, genome, genomic variation, expression, GO, homologous genes and several pieces of literature. It provides a public platform for online data analysis, visualization, and downloading of results. All the data and the incorporated tools for online analysis and visualization make iDog a valuable resource for the dog research community with a wider appeal to dog owners or veterinarians. In the future, we will keep updating the existing dataset as new data of high quality is made available. For instance, we plan to integrate the standard reference genome (Canfam3.1 or a newer version) in DogGD, host more individuals and additional SNPs when the more data are generated under the dog10k project, incorporate more paleogenomic data (43,44) into the DogSD, collect more phenotypic information, especially quantitative data, to expand the DogPD and include more expression data for both coding or non-coding genes (45,46) into the DogED. Moreover, we will continue developing and integrating more tools for genomes, population, evolution and network analysis, as well as more interactive visualization methods for various omics data. We also welcome comments and suggestions from researchers all over the world to improve iDog.
